# 5,6-Dimeth­oxy-4′,5′-diphenyl­indane-2-spiro-3′-pyrrolidine-2′-spiro-3′′-indoline-1,2′′-dione

**DOI:** 10.1107/S1600536810035865

**Published:** 2010-09-11

**Authors:** Mohamed Ashraf Ali, Rusli Ismail, Soo Choon Tan, Chin Sing Yeap, Hoong-Kun Fun

**Affiliations:** a Institute for Research in Molecular Medicine, Universiti Sains Malaysia, 11800 USM, Penang, Malaysia; b X-ray Crystallography Unit, School of Physics, Universiti Sains Malaysia, 11800 USM, Penang, Malaysia

## Abstract

In the title compound, C_33_H_28_N_2_O_4_, the central pyrrolidine ring adopts a half-chair conformation. Both the indolinone and indanone groups are twisted, with their five-membered rings adopting a half-chair and an envelope conformation, respectively. The two benzene rings and the mean plane of the indolinone and indanone groups make dihedral angles of 71.98 (10), 84.32 (10), 86.26 (9) and 78.50 (9)°, respectively, with the central pyrrolidine ring. Intra­molecular C—H⋯O hydrogen bonds stabilize the mol­ecular conformation. In the crystal, pairs of inter­molecular N—H⋯O hydrogen bonds link the mol­ecules into centrosymmetric dimers. The dimers are inter­connected into ribbons propagating along [110] *via* weak inter­molecular C—H⋯O hydrogen bonds. Weak inter­molecular C—H⋯π and π–π [centroid–centroid distance = 3.6509 (11) Å] inter­actions are also observed.

## Related literature

For general background to heterocycles, see: Kirsch *et al.* (2004 ▶); Shi *et al.* (2009 ▶); Nair *et al.* (2007 ▶); Nájera *et al.* (2005 ▶); Coldham *et al.* (2005 ▶). For general background to pyrrolidine derivatives, see: Daly *et al.* (1986 ▶). For the biological activity of isatin derivatives and spiro­pyrrolidinyloxindoles, see: Cui *et al.* (1996 ▶); Xue *et al.* (2000 ▶); Klumpp *et al.* (1998 ▶); Hilton *et al.* (2000 ▶). For ring conformations, see Cremer & Pople (1975 ▶). For the stability of the temperature controller used in the data collection, see: Cosier & Glazer (1986 ▶).
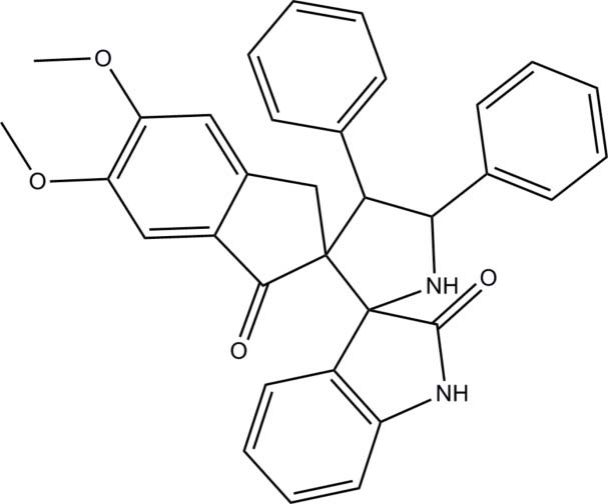


## Experimental

### 

#### Crystal data

C_33_H_28_N_2_O_4_
*M* *_r_* = 516.57
Triclinic, 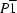

*a* = 9.2746 (12) Å
*b* = 10.6337 (15) Å
*c* = 14.4279 (19) Å
α = 92.369 (3)°
β = 98.557 (3)°
γ = 115.341 (2)°
*V* = 1262.9 (3) Å^3^
*Z* = 2
Mo *K*α radiation
μ = 0.09 mm^−1^
*T* = 100 K
0.28 × 0.19 × 0.07 mm

#### Data collection

Bruker APEXII DUO CCD area-detector diffractometer
Absorption correction: multi-scan (*SADABS*; Bruker, 2009 ▶) *T*_min_ = 0.976, *T*_max_ = 0.994
20001 measured reflections
7399 independent reflections
4778 reflections with *I* > 2σ(*I*)
*R*_int_ = 0.054

#### Refinement

*R*[*F* ^2^ > 2σ(*F* ^2^)] = 0.056
*wR*(*F* ^2^) = 0.159
*S* = 1.05
7399 reflections
360 parameters
H atoms treated by a mixture of independent and constrained refinement
Δρ_max_ = 0.41 e Å^−3^
Δρ_min_ = −0.32 e Å^−3^

### 

Data collection: *APEX2* (Bruker, 2009 ▶); cell refinement: *SAINT* (Bruker, 2009 ▶); data reduction: *SAINT*; program(s) used to solve structure: *SHELXTL* (Sheldrick, 2008 ▶); program(s) used to refine structure: *SHELXTL*; molecular graphics: *SHELXTL*; software used to prepare material for publication: *SHELXTL* and *PLATON* (Spek, 2009 ▶).

## Supplementary Material

Crystal structure: contains datablocks global, I. DOI: 10.1107/S1600536810035865/rz2484sup1.cif

Structure factors: contains datablocks I. DOI: 10.1107/S1600536810035865/rz2484Isup2.hkl

Additional supplementary materials:  crystallographic information; 3D view; checkCIF report

## Figures and Tables

**Table 1:** Hydrogen-bond geometry (Å, °) *Cg*2, *Cg*3 and *Cg*4 are the centroids of the C26–C31, C20–C25 and C13–C18 benzene rings, respectively.

*D*—H⋯*A*	*D*—H	H⋯*A*	*D*⋯*A*	*D*—H⋯*A*
N2—H1*N*2⋯O2^i^	0.94 (2)	1.94 (2)	2.8559 (19)	164 (2)
C8—H8*B*⋯O2	0.97	2.49	3.214 (2)	131
C11—H11*A*⋯O2	0.98	2.55	3.148 (2)	119
C22—H22*A*⋯O4^ii^	0.93	2.59	3.471 (2)	159
N1—H1*N*1⋯*Cg*2^iii^	0.93 (2)	2.55 (2)	3.4518 (17)	161.7 (16)
C17—H17*A*⋯*Cg*3^iv^	0.93	2.81	3.5609 (18)	139
C28—H28*A*⋯*Cg*4^iii^	0.93	2.92	3.501 (2)	121

Symmetry codes: (i) 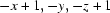; (ii) 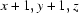; (iii) 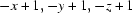; (iv) 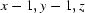.

**Fig. 1.**
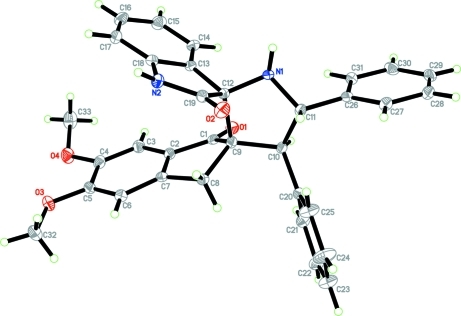
The molecular structure of the title compound with atom labels and 50% probability ellipsoids for non-H atoms.

**Fig. 2.**
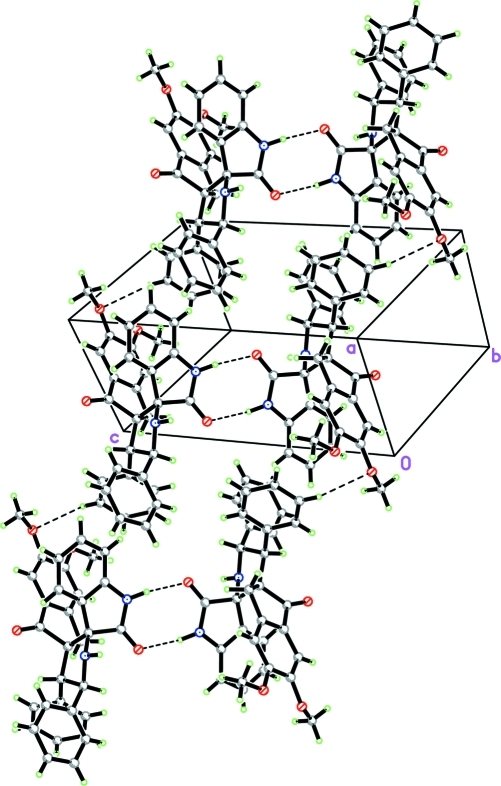
The crystal packing of title compound, showing chains along the [110] direction. Intermolecular hydrogen bonds are shown as dashed lines.

## supplementary crystallographic information

### Comment

The development of new efficient methods to synthesize nitrogen heterocycles with structural diversity is one of the major objectives of modern synthetic organic chemists (Kirsch *et al.*, 2004). Multicomponent 1,3-dipolar cycloaddition of ylidic species, such as azomethine ylides with olefinic dipolarophiles, plays a key role in the construction of biologically active five-membered heterocycles (Shi *et al.*, 2009; Nair *et al.*, 2007; Nájera *et al.*, 2005; Coldham *et al.*, 2005). Highly substituted pyrrolidines have gained much prominence since they form the central skeleton of many natural products (Daly *et al.*, 1986). Isatin and its derivatives possess interesting biological activities and are widely used as precursors for many natural products (Cui *et al.*, 1996, Xue *et al.*, 2000, Klumpp *et al.*, 1998). Spiropyrrolidinyloxindoles are also found in a number of alkaloids of biological importance (Hilton *et al.*, 2000). Due to the biological importance of the aforesaid heterocycles, the crystal structure determination of the title compound was carried out and the results are presented in this paper.

The molecular structure of the title compound is shown in Fig. 1. The central pyrrolidine ring (N1/C12/C9–C11) adopts a half-chair conformation (twisted on the N1–C11 bond), with puckering parameters Q = 0.4196 (18) Å and φ = 198.1 (2)° (Cremer & Pople, 1975). Both indolinone and indanone groups are twisted with their five-membered rings adopting a half chair (twisted on the C12–C19 bond) and an envelope (flap on the C9 atom) conformation respectively. The puckering parameters of these two rings are Q = 0.1316 (18) Å, φ = 127.6 (8)° and Q = 0.2814 (19) Å, φ = 323.9 (4)°. The two benzene rings (C20–C25 and C26–C31) and the mean plane of indolinone and indanone groups make dihedral angles of 71.98 (10), 84.32 (10), 86.26 (9) and 78.50 (9)°, respectively, with the central pyrrolidine ring. Intramolecular C8—H8B···O2 and C11—H11A···O2 hydrogen bonds (Table 1) stabilize the molecular structure. In the crystal structure, intermolecular N2—H1N2···O2 hydrogen bonds (Table 1) link the molecules into centrosymmetric dimers (Fig. 2). These dimers are interconnected into ribbons propagating along the [110] direction *via* weak intermolecular C22—H22A···O4 hydrogen bonds (Fig. 2, Table 1). Weak intermolecular C—H···π (Table 1) and π–π interactions are also observed (*Cg*1···*Cg*1^v^ = 3.6509 (11) Å; (v) = 1-*x*, -*y*, -*z. Cg*1 is centroid of benzene ring C2–C7).

### Experimental

A mixture of (*E*)-2-bezylylidene-5,6-dimethoxy-2,3-dihydro-1*H*-indene-1-one (0.001 mmol), isatin (0.001 mmol) and phenylglycine (0.002 mmol) was dissolved in methanol (10 ml) and refluxed for 4 h. After completion of the reaction as evident from TLC, the mixture was poured into water (50 ml). The precipitated solid was filtered, washed with water and recrystallized from a petroleum ether-ethyl acetate mixture (1:1 *v*/*v*) to give the title compound as yellow crystals.

### Refinement

The N-bound hydrogen atoms were located from the difference Fourier map and refined freely. All other hydrogen atoms were positioned geometrically and refined using a riding model, with C—H = 0.93–0.98 Å, and with *U*_iso_(H) = 1.2 *U*_eq_(C) or 1.5 *U*_eq_(C) for methyl H atoms. A rotating-group model were applied for the methyl groups.

### Crystal data

**Table:**

C_33_H_28_N_2_O_4_	*Z* = 2
*M**_r_* = 516.57	*F*(000) = 544
Triclinic, *P*1	*D*_x_ = 1.358 Mg m^−^^3^
Hall symbol: -P 1	Mo *K*α radiation, λ = 0.71073 Å
*a* = 9.2746 (12) Å	Cell parameters from 2918 reflections
*b* = 10.6337 (15) Å	θ = 2.4–29.7°
*c* = 14.4279 (19) Å	µ = 0.09 mm^−^^1^
α = 92.369 (3)°	*T* = 100 K
β = 98.557 (3)°	Plate, yellow
γ = 115.341 (2)°	0.28 × 0.19 × 0.07 mm
*V* = 1262.9 (3) Å^3^	

### Data collection

**Table:**

Bruker APEXII DUO CCD area-detector diffractometer	7399 independent reflections
Radiation source: fine-focus sealed tube	4778 reflections with *I* > 2σ(*I*)
graphite	*R*_int_ = 0.054
φ and ω scans	θ_max_ = 30.2°, θ_min_ = 1.4°
Absorption correction: multi-scan (*SADABS*; Bruker, 2009)	*h* = −13→13
*T*_min_ = 0.976, *T*_max_ = 0.994	*k* = −15→15
20001 measured reflections	*l* = −20→20

### Refinement

**Table:**

Refinement on *F*^2^	Primary atom site location: structure-invariant direct methods
Least-squares matrix: full	Secondary atom site location: difference Fourier map
*R*[*F*^2^ > 2σ(*F*^2^)] = 0.056	Hydrogen site location: inferred from neighbouring sites
*wR*(*F*^2^) = 0.159	H atoms treated by a mixture of independent and constrained refinement
*S* = 1.05	*w* = 1/[σ^2^(*F*_o_^2^) + (0.0759*P*)^2^ + 0.0467*P*] where *P* = (*F*_o_^2^ + 2*F*_c_^2^)/3
7399 reflections	(Δ/σ)_max_ < 0.001
360 parameters	Δρ_max_ = 0.41 e Å^−^^3^
0 restraints	Δρ_min_ = −0.32 e Å^−^^3^

### Special details

**Table:**

Experimental. The crystal was placed in the cold stream of an Oxford Cryosystems Cobra open-flow nitrogen cryostat (Cosier & Glazer, 1986) operating at 100.0 (1) K.
Geometry. All e.s.d.'s (except the e.s.d. in the dihedral angle between two l.s. planes) are estimated using the full covariance matrix. The cell e.s.d.'s are taken into account individually in the estimation of e.s.d.'s in distances, angles and torsion angles; correlations between e.s.d.'s in cell parameters are only used when they are defined by crystal symmetry. An approximate (isotropic) treatment of cell e.s.d.'s is used for estimating e.s.d.'s involving l.s. planes.
Refinement. Refinement of *F*^2^ against ALL reflections. The weighted *R*-factor *wR* and goodness of fit *S* are based on *F*^2^, conventional *R*-factors *R* are based on *F*, with *F* set to zero for negative *F*^2^. The threshold expression of *F*^2^ > σ(*F*^2^) is used only for calculating *R*-factors(gt) *etc*. and is not relevant to the choice of reflections for refinement. *R*-factors based on *F*^2^ are statistically about twice as large as those based on *F*, and *R*- factors based on ALL data will be even larger.

### Fractional atomic coordinates and isotropic or equivalent isotropic displacement parameters (Å^2^)

**Table:**

	*x*	*y*	*z*	*U*_iso_*/*U*_eq_	
O1	0.38580 (14)	0.30325 (11)	0.12688 (8)	0.0175 (3)	
O2	0.63136 (15)	0.17078 (11)	0.45955 (9)	0.0190 (3)	
O3	0.36325 (15)	−0.30867 (11)	0.04324 (9)	0.0220 (3)	
O4	0.13257 (16)	−0.24781 (12)	−0.02810 (9)	0.0235 (3)	
N1	0.47665 (17)	0.35081 (13)	0.37988 (10)	0.0135 (3)	
N2	0.38428 (18)	−0.00712 (13)	0.38690 (10)	0.0164 (3)	
C1	0.4391 (2)	0.22526 (15)	0.16088 (12)	0.0138 (3)	
C2	0.4020 (2)	0.08196 (15)	0.12244 (12)	0.0147 (3)	
C3	0.2695 (2)	−0.01163 (16)	0.05522 (12)	0.0172 (4)	
H3A	0.1907	0.0138	0.0268	0.021*	
C4	0.2594 (2)	−0.14310 (16)	0.03240 (12)	0.0178 (4)	
C5	0.3844 (2)	−0.17795 (16)	0.07342 (12)	0.0170 (4)	
C6	0.5147 (2)	−0.08336 (16)	0.14044 (12)	0.0172 (4)	
H6A	0.5966	−0.1062	0.1672	0.021*	
C7	0.5197 (2)	0.04618 (15)	0.16652 (12)	0.0142 (3)	
C8	0.6448 (2)	0.16427 (15)	0.23820 (12)	0.0150 (3)	
H8A	0.7433	0.2149	0.2136	0.018*	
H8B	0.6715	0.1293	0.2961	0.018*	
C9	0.5599 (2)	0.25859 (15)	0.25492 (11)	0.0133 (3)	
C10	0.66796 (19)	0.41814 (14)	0.28257 (11)	0.0129 (3)	
H10A	0.6179	0.4660	0.2422	0.016*	
C11	0.6481 (2)	0.44920 (15)	0.38374 (12)	0.0136 (3)	
H11A	0.7192	0.4258	0.4297	0.016*	
C12	0.44986 (19)	0.21556 (15)	0.33531 (11)	0.0128 (3)	
C13	0.2716 (2)	0.11722 (15)	0.30263 (11)	0.0134 (3)	
C14	0.1448 (2)	0.14017 (17)	0.25703 (13)	0.0177 (4)	
H14A	0.1620	0.2282	0.2401	0.021*	
C15	−0.0105 (2)	0.02774 (18)	0.23695 (13)	0.0225 (4)	
H15A	−0.0973	0.0411	0.2060	0.027*	
C16	−0.0361 (2)	−0.10321 (18)	0.26268 (14)	0.0228 (4)	
H16A	−0.1395	−0.1772	0.2469	0.027*	
C17	0.0892 (2)	−0.12627 (16)	0.31155 (13)	0.0200 (4)	
H17A	0.0716	−0.2137	0.3300	0.024*	
C18	0.2417 (2)	−0.01366 (16)	0.33174 (12)	0.0152 (3)	
C19	0.5038 (2)	0.12758 (15)	0.40229 (12)	0.0150 (3)	
C20	0.8407 (2)	0.47122 (15)	0.26691 (12)	0.0144 (3)	
C21	0.8771 (2)	0.51532 (16)	0.17995 (12)	0.0177 (4)	
H21A	0.7952	0.5145	0.1337	0.021*	
C22	1.0331 (2)	0.56052 (17)	0.16100 (13)	0.0215 (4)	
H22A	1.0546	0.5885	0.1023	0.026*	
C23	1.1560 (2)	0.56373 (19)	0.22949 (15)	0.0265 (4)	
H23A	1.2609	0.5954	0.2174	0.032*	
C24	1.1231 (2)	0.5198 (2)	0.31617 (15)	0.0302 (5)	
H24A	1.2057	0.5213	0.3621	0.036*	
C25	0.9666 (2)	0.47344 (19)	0.33457 (14)	0.0230 (4)	
H25A	0.9454	0.4434	0.3928	0.028*	
C26	0.6764 (2)	0.59922 (15)	0.40689 (12)	0.0141 (3)	
C27	0.8097 (2)	0.69237 (16)	0.47249 (12)	0.0175 (4)	
H27A	0.8812	0.6619	0.5047	0.021*	
C28	0.8366 (2)	0.83123 (16)	0.49029 (13)	0.0199 (4)	
H28A	0.9251	0.8926	0.5350	0.024*	
C29	0.7325 (2)	0.87811 (16)	0.44184 (13)	0.0209 (4)	
H29A	0.7525	0.9715	0.4526	0.025*	
C30	0.5976 (2)	0.78502 (17)	0.37694 (13)	0.0202 (4)	
H30A	0.5261	0.8157	0.3450	0.024*	
C31	0.5697 (2)	0.64619 (16)	0.35990 (12)	0.0166 (4)	
H31A	0.4791	0.5841	0.3168	0.020*	
C32	0.4881 (2)	−0.34828 (18)	0.07941 (14)	0.0247 (4)	
H32A	0.4591	−0.4421	0.0529	0.037*	
H32B	0.5002	−0.3441	0.1469	0.037*	
H32C	0.5886	−0.2853	0.0627	0.037*	
C33	−0.0201 (2)	−0.24280 (19)	−0.03764 (15)	0.0283 (5)	
H33A	−0.0999	−0.3202	−0.0815	0.042*	
H33B	−0.0102	−0.1565	−0.0605	0.042*	
H33C	−0.0531	−0.2484	0.0226	0.042*	
H1N1	0.444 (2)	0.3484 (19)	0.4383 (14)	0.017 (5)*	
H1N2	0.380 (3)	−0.073 (2)	0.4293 (18)	0.047 (7)*	

### Atomic displacement parameters (Å^2^)

**Table:**

	*U*^11^	*U*^22^	*U*^33^	*U*^12^	*U*^13^	*U*^23^
O1	0.0181 (7)	0.0174 (5)	0.0193 (6)	0.0094 (5)	0.0047 (5)	0.0057 (4)
O2	0.0173 (7)	0.0188 (5)	0.0199 (6)	0.0072 (5)	0.0021 (5)	0.0052 (5)
O3	0.0247 (7)	0.0154 (5)	0.0263 (7)	0.0094 (5)	0.0056 (6)	−0.0030 (5)
O4	0.0218 (7)	0.0198 (6)	0.0229 (7)	0.0061 (5)	−0.0019 (6)	−0.0054 (5)
N1	0.0127 (7)	0.0118 (5)	0.0167 (7)	0.0046 (5)	0.0074 (6)	0.0016 (5)
N2	0.0179 (8)	0.0131 (6)	0.0185 (7)	0.0065 (5)	0.0047 (6)	0.0041 (5)
C1	0.0124 (8)	0.0148 (6)	0.0152 (8)	0.0056 (6)	0.0059 (7)	0.0043 (6)
C2	0.0169 (9)	0.0130 (6)	0.0143 (8)	0.0057 (6)	0.0057 (7)	0.0028 (6)
C3	0.0189 (9)	0.0161 (7)	0.0157 (8)	0.0067 (6)	0.0039 (7)	0.0023 (6)
C4	0.0182 (9)	0.0163 (7)	0.0141 (8)	0.0033 (6)	0.0027 (7)	−0.0005 (6)
C5	0.0205 (9)	0.0140 (7)	0.0171 (8)	0.0070 (6)	0.0079 (7)	0.0009 (6)
C6	0.0170 (9)	0.0157 (7)	0.0198 (9)	0.0071 (6)	0.0059 (7)	0.0016 (6)
C7	0.0149 (9)	0.0141 (6)	0.0146 (8)	0.0056 (6)	0.0075 (7)	0.0027 (6)
C8	0.0124 (8)	0.0140 (6)	0.0192 (8)	0.0057 (6)	0.0049 (7)	0.0023 (6)
C9	0.0133 (8)	0.0115 (6)	0.0145 (8)	0.0047 (6)	0.0027 (7)	0.0022 (5)
C10	0.0118 (8)	0.0111 (6)	0.0153 (8)	0.0043 (6)	0.0029 (7)	0.0022 (5)
C11	0.0116 (8)	0.0121 (6)	0.0159 (8)	0.0044 (6)	0.0019 (7)	0.0018 (5)
C12	0.0118 (8)	0.0118 (6)	0.0152 (8)	0.0050 (6)	0.0043 (7)	0.0023 (5)
C13	0.0123 (8)	0.0130 (6)	0.0138 (8)	0.0041 (6)	0.0046 (7)	0.0011 (5)
C14	0.0136 (9)	0.0182 (7)	0.0217 (9)	0.0063 (6)	0.0066 (7)	0.0027 (6)
C15	0.0159 (10)	0.0246 (8)	0.0242 (10)	0.0069 (7)	0.0023 (8)	0.0014 (7)
C16	0.0130 (9)	0.0215 (8)	0.0271 (10)	0.0012 (6)	0.0052 (8)	−0.0016 (7)
C17	0.0215 (10)	0.0133 (7)	0.0222 (9)	0.0034 (6)	0.0087 (8)	0.0011 (6)
C18	0.0151 (9)	0.0155 (7)	0.0158 (8)	0.0067 (6)	0.0058 (7)	0.0014 (6)
C19	0.0170 (9)	0.0133 (6)	0.0168 (8)	0.0073 (6)	0.0065 (7)	0.0034 (6)
C20	0.0131 (8)	0.0116 (6)	0.0182 (8)	0.0045 (6)	0.0050 (7)	0.0015 (6)
C21	0.0167 (9)	0.0184 (7)	0.0172 (8)	0.0070 (6)	0.0034 (7)	0.0030 (6)
C22	0.0200 (10)	0.0230 (8)	0.0214 (9)	0.0073 (7)	0.0093 (8)	0.0061 (7)
C23	0.0175 (10)	0.0316 (9)	0.0305 (11)	0.0087 (7)	0.0103 (9)	0.0082 (8)
C24	0.0156 (10)	0.0464 (11)	0.0295 (11)	0.0134 (8)	0.0048 (9)	0.0137 (9)
C25	0.0162 (10)	0.0321 (9)	0.0238 (10)	0.0113 (7)	0.0078 (8)	0.0125 (7)
C26	0.0142 (9)	0.0131 (6)	0.0159 (8)	0.0054 (6)	0.0071 (7)	0.0022 (6)
C27	0.0159 (9)	0.0161 (7)	0.0189 (8)	0.0051 (6)	0.0048 (7)	0.0028 (6)
C28	0.0191 (10)	0.0152 (7)	0.0204 (9)	0.0018 (6)	0.0075 (7)	0.0005 (6)
C29	0.0273 (10)	0.0137 (7)	0.0237 (9)	0.0075 (7)	0.0150 (8)	0.0031 (6)
C30	0.0236 (10)	0.0201 (7)	0.0230 (9)	0.0138 (7)	0.0082 (8)	0.0049 (6)
C31	0.0160 (9)	0.0150 (7)	0.0188 (8)	0.0064 (6)	0.0049 (7)	0.0010 (6)
C32	0.0298 (11)	0.0196 (8)	0.0299 (10)	0.0142 (7)	0.0101 (9)	0.0022 (7)
C33	0.0230 (11)	0.0236 (8)	0.0314 (11)	0.0083 (7)	−0.0082 (9)	−0.0013 (7)

### Geometric parameters (Å, °)

**Table:**

O1—C1	1.2189 (19)	C14—C15	1.400 (2)
O2—C19	1.227 (2)	C14—H14A	0.9300
O3—C5	1.3606 (18)	C15—C16	1.385 (3)
O3—C32	1.430 (2)	C15—H15A	0.9300
O4—C4	1.3704 (19)	C16—C17	1.387 (3)
O4—C33	1.427 (2)	C16—H16A	0.9300
N1—C12	1.4540 (19)	C17—C18	1.385 (2)
N1—C11	1.473 (2)	C17—H17A	0.9300
N1—H1N1	0.93 (2)	C20—C21	1.395 (2)
N2—C19	1.3678 (19)	C20—C25	1.398 (2)
N2—C18	1.411 (2)	C21—C22	1.390 (3)
N2—H1N2	0.94 (3)	C21—H21A	0.9300
C1—C2	1.474 (2)	C22—C23	1.381 (3)
C1—C9	1.546 (2)	C22—H22A	0.9300
C2—C7	1.382 (2)	C23—C24	1.384 (3)
C2—C3	1.400 (2)	C23—H23A	0.9300
C3—C4	1.383 (2)	C24—C25	1.390 (3)
C3—H3A	0.9300	C24—H24A	0.9300
C4—C5	1.419 (3)	C25—H25A	0.9300
C5—C6	1.392 (2)	C26—C27	1.390 (2)
C6—C7	1.392 (2)	C26—C31	1.393 (2)
C6—H6A	0.9300	C27—C28	1.394 (2)
C7—C8	1.510 (2)	C27—H27A	0.9300
C8—C9	1.548 (2)	C28—C29	1.382 (3)
C8—H8A	0.9700	C28—H28A	0.9300
C8—H8B	0.9700	C29—C30	1.392 (2)
C9—C10	1.5529 (19)	C29—H29A	0.9300
C9—C12	1.610 (2)	C30—C31	1.390 (2)
C10—C20	1.510 (2)	C30—H30A	0.9300
C10—C11	1.538 (2)	C31—H31A	0.9300
C10—H10A	0.9800	C32—H32A	0.9600
C11—C26	1.515 (2)	C32—H32B	0.9600
C11—H11A	0.9800	C32—H32C	0.9600
C12—C13	1.515 (2)	C33—H33A	0.9600
C12—C19	1.546 (2)	C33—H33B	0.9600
C13—C14	1.380 (2)	C33—H33C	0.9600
C13—C18	1.394 (2)		
			
C5—O3—C32	117.45 (13)	C15—C14—H14A	120.8
C4—O4—C33	116.29 (14)	C16—C15—C14	120.72 (18)
C12—N1—C11	107.96 (13)	C16—C15—H15A	119.6
C12—N1—H1N1	114.7 (11)	C14—C15—H15A	119.6
C11—N1—H1N1	113.2 (11)	C15—C16—C17	121.36 (16)
C19—N2—C18	110.48 (13)	C15—C16—H16A	119.3
C19—N2—H1N2	122.2 (14)	C17—C16—H16A	119.3
C18—N2—H1N2	121.4 (14)	C18—C17—C16	117.18 (15)
O1—C1—C2	127.74 (15)	C18—C17—H17A	121.4
O1—C1—C9	125.95 (14)	C16—C17—H17A	121.4
C2—C1—C9	106.31 (13)	C17—C18—C13	122.37 (16)
C7—C2—C3	122.17 (14)	C17—C18—N2	127.89 (15)
C7—C2—C1	108.94 (14)	C13—C18—N2	109.63 (14)
C3—C2—C1	128.85 (16)	O2—C19—N2	125.76 (15)
C4—C3—C2	117.96 (16)	O2—C19—C12	126.09 (14)
C4—C3—H3A	121.0	N2—C19—C12	108.15 (14)
C2—C3—H3A	121.0	C21—C20—C25	117.69 (16)
O4—C4—C3	124.41 (16)	C21—C20—C10	119.25 (15)
O4—C4—C5	115.38 (14)	C25—C20—C10	123.00 (16)
C3—C4—C5	120.21 (15)	C22—C21—C20	121.40 (17)
O3—C5—C6	124.50 (16)	C22—C21—H21A	119.3
O3—C5—C4	114.75 (14)	C20—C21—H21A	119.3
C6—C5—C4	120.74 (14)	C23—C22—C21	119.83 (18)
C5—C6—C7	118.61 (16)	C23—C22—H22A	120.1
C5—C6—H6A	120.7	C21—C22—H22A	120.1
C7—C6—H6A	120.7	C22—C23—C24	119.96 (19)
C2—C7—C6	120.14 (15)	C22—C23—H23A	120.0
C2—C7—C8	111.20 (13)	C24—C23—H23A	120.0
C6—C7—C8	128.63 (16)	C23—C24—C25	120.06 (19)
C7—C8—C9	103.46 (13)	C23—C24—H24A	120.0
C7—C8—H8A	111.1	C25—C24—H24A	120.0
C9—C8—H8A	111.1	C24—C25—C20	121.04 (18)
C7—C8—H8B	111.1	C24—C25—H25A	119.5
C9—C8—H8B	111.1	C20—C25—H25A	119.5
H8A—C8—H8B	109.0	C27—C26—C31	119.13 (14)
C1—C9—C8	102.10 (13)	C27—C26—C11	121.23 (15)
C1—C9—C10	112.78 (12)	C31—C26—C11	119.62 (14)
C8—C9—C10	117.99 (14)	C26—C27—C28	120.29 (17)
C1—C9—C12	105.46 (13)	C26—C27—H27A	119.9
C8—C9—C12	114.60 (12)	C28—C27—H27A	119.9
C10—C9—C12	103.53 (12)	C29—C28—C27	120.33 (16)
C20—C10—C11	115.67 (13)	C29—C28—H28A	119.8
C20—C10—C9	115.57 (13)	C27—C28—H28A	119.8
C11—C10—C9	105.43 (12)	C28—C29—C30	119.71 (15)
C20—C10—H10A	106.5	C28—C29—H29A	120.1
C11—C10—H10A	106.5	C30—C29—H29A	120.1
C9—C10—H10A	106.5	C31—C30—C29	119.96 (17)
N1—C11—C26	111.09 (14)	C31—C30—H30A	120.0
N1—C11—C10	100.59 (12)	C29—C30—H30A	120.0
C26—C11—C10	112.87 (13)	C30—C31—C26	120.55 (16)
N1—C11—H11A	110.6	C30—C31—H31A	119.7
C26—C11—H11A	110.6	C26—C31—H31A	119.7
C10—C11—H11A	110.6	O3—C32—H32A	109.5
N1—C12—C13	112.92 (14)	O3—C32—H32B	109.5
N1—C12—C19	114.28 (13)	H32A—C32—H32B	109.5
C13—C12—C19	100.96 (12)	O3—C32—H32C	109.5
N1—C12—C9	102.56 (12)	H32A—C32—H32C	109.5
C13—C12—C9	116.62 (13)	H32B—C32—H32C	109.5
C19—C12—C9	110.00 (13)	O4—C33—H33A	109.5
C14—C13—C18	119.81 (15)	O4—C33—H33B	109.5
C14—C13—C12	131.21 (14)	H33A—C33—H33B	109.5
C18—C13—C12	108.86 (14)	O4—C33—H33C	109.5
C13—C14—C15	118.43 (15)	H33A—C33—H33C	109.5
C13—C14—H14A	120.8	H33B—C33—H33C	109.5
			
O1—C1—C2—C7	162.86 (17)	C10—C9—C12—C13	137.35 (14)
C9—C1—C2—C7	−17.63 (18)	C1—C9—C12—C19	132.81 (13)
O1—C1—C2—C3	−19.4 (3)	C8—C9—C12—C19	21.35 (17)
C9—C1—C2—C3	160.08 (17)	C10—C9—C12—C19	−108.52 (13)
C7—C2—C3—C4	−0.8 (3)	N1—C12—C13—C14	42.9 (2)
C1—C2—C3—C4	−178.21 (17)	C19—C12—C13—C14	165.38 (18)
C33—O4—C4—C3	−25.8 (3)	C9—C12—C13—C14	−75.5 (2)
C33—O4—C4—C5	153.32 (16)	N1—C12—C13—C18	−132.94 (15)
C2—C3—C4—O4	176.31 (16)	C19—C12—C13—C18	−10.49 (17)
C2—C3—C4—C5	−2.8 (3)	C9—C12—C13—C18	108.63 (16)
C32—O3—C5—C6	−3.4 (3)	C18—C13—C14—C15	−3.3 (3)
C32—O3—C5—C4	177.80 (15)	C12—C13—C14—C15	−178.79 (17)
O4—C4—C5—O3	2.8 (2)	C13—C14—C15—C16	0.3 (3)
C3—C4—C5—O3	−178.02 (16)	C14—C15—C16—C17	2.1 (3)
O4—C4—C5—C6	−176.01 (16)	C15—C16—C17—C18	−1.3 (3)
C3—C4—C5—C6	3.2 (3)	C16—C17—C18—C13	−1.8 (3)
O3—C5—C6—C7	−178.60 (16)	C16—C17—C18—N2	174.15 (17)
C4—C5—C6—C7	0.1 (3)	C14—C13—C18—C17	4.1 (3)
C3—C2—C7—C6	4.0 (3)	C12—C13—C18—C17	−179.45 (16)
C1—C2—C7—C6	−178.06 (15)	C14—C13—C18—N2	−172.44 (16)
C3—C2—C7—C8	−177.88 (16)	C12—C13—C18—N2	3.98 (19)
C1—C2—C7—C8	0.0 (2)	C19—N2—C18—C17	−170.80 (18)
C5—C6—C7—C2	−3.6 (3)	C19—N2—C18—C13	5.5 (2)
C5—C6—C7—C8	178.69 (16)	C18—N2—C19—O2	167.44 (17)
C2—C7—C8—C9	17.35 (18)	C18—N2—C19—C12	−12.43 (19)
C6—C7—C8—C9	−164.79 (17)	N1—C12—C19—O2	−44.6 (2)
O1—C1—C9—C8	−153.34 (17)	C13—C12—C19—O2	−166.11 (17)
C2—C1—C9—C8	27.14 (16)	C9—C12—C19—O2	70.1 (2)
O1—C1—C9—C10	−25.7 (2)	N1—C12—C19—N2	135.26 (15)
C2—C1—C9—C10	154.77 (14)	C13—C12—C19—N2	13.76 (17)
O1—C1—C9—C12	86.59 (19)	C9—C12—C19—N2	−110.03 (15)
C2—C1—C9—C12	−92.93 (14)	C11—C10—C20—C21	−146.90 (14)
C7—C8—C9—C1	−26.21 (15)	C9—C10—C20—C21	89.24 (18)
C7—C8—C9—C10	−150.44 (14)	C11—C10—C20—C25	35.7 (2)
C7—C8—C9—C12	87.24 (15)	C9—C10—C20—C25	−88.16 (19)
C1—C9—C10—C20	−104.01 (17)	C25—C20—C21—C22	−0.2 (2)
C8—C9—C10—C20	14.7 (2)	C10—C20—C21—C22	−177.77 (14)
C12—C9—C10—C20	142.51 (14)	C20—C21—C22—C23	−0.7 (3)
C1—C9—C10—C11	126.92 (15)	C21—C22—C23—C24	1.1 (3)
C8—C9—C10—C11	−114.35 (16)	C22—C23—C24—C25	−0.5 (3)
C12—C9—C10—C11	13.44 (16)	C23—C24—C25—C20	−0.5 (3)
C12—N1—C11—C26	166.16 (13)	C21—C20—C25—C24	0.8 (3)
C12—N1—C11—C10	46.44 (15)	C10—C20—C25—C24	178.29 (16)
C20—C10—C11—N1	−164.04 (12)	N1—C11—C26—C27	135.61 (16)
C9—C10—C11—N1	−35.03 (15)	C10—C11—C26—C27	−112.28 (18)
C20—C10—C11—C26	77.52 (17)	N1—C11—C26—C31	−46.1 (2)
C9—C10—C11—C26	−153.47 (14)	C10—C11—C26—C31	66.0 (2)
C11—N1—C12—C13	−164.04 (13)	C31—C26—C27—C28	−0.5 (3)
C11—N1—C12—C19	81.30 (17)	C11—C26—C27—C28	177.73 (16)
C11—N1—C12—C9	−37.70 (16)	C26—C27—C28—C29	−1.0 (3)
C1—C9—C12—N1	−105.23 (13)	C27—C28—C29—C30	1.8 (3)
C8—C9—C12—N1	143.31 (13)	C28—C29—C30—C31	−1.1 (3)
C10—C9—C12—N1	13.44 (15)	C29—C30—C31—C26	−0.4 (3)
C1—C9—C12—C13	18.68 (17)	C27—C26—C31—C30	1.2 (3)
C8—C9—C12—C13	−92.78 (17)	C11—C26—C31—C30	−177.07 (16)

### Hydrogen-bond geometry (Å, °)

**Table:**

Cg2, Cg3 and Cg4 are the centroids of the C26–C31, C20–C25 and C13–C18 benzene rings, respectively.

**Table:**

*D*—H···*A*	*D*—H	H···*A*	*D*···*A*	*D*—H···*A*
N2—H1N2···O2^i^	0.94 (2)	1.94 (2)	2.8559 (19)	164 (2)
C8—H8B···O2	0.97	2.49	3.214 (2)	131
C11—H11A···O2	0.98	2.55	3.148 (2)	119
C22—H22A···O4^ii^	0.93	2.59	3.471 (2)	159
N1—H1N1···Cg2^iii^	0.93 (2)	2.55 (2)	3.4518 (17)	161.7 (16)
C17—H17A···Cg3^iv^	0.93	2.81	3.5609 (18)	139
C28—H28A···Cg4^iii^	0.93	2.92	3.501 (2)	121

Symmetry codes: (i) −*x*+1, −*y*, −*z*+1; (ii) *x*+1, *y*+1, *z*; (iii) −*x*+1, −*y*+1, −*z*+1; (iv) *x*−1, *y*−1, *z*.
